# Simulated tri-trophic networks reveal complex relationships between species diversity and interaction diversity

**DOI:** 10.1371/journal.pone.0193822

**Published:** 2018-03-26

**Authors:** Nicholas A. Pardikes, Will Lumpkin, Paul J. Hurtado, Lee A. Dyer

**Affiliations:** 1 Department of Biology, Program in Ecology, Evolution, and Conservation Biology, University of Nevada, Reno, Nevada, United States of America; 2 Czech Academy of Sciences, Institute of Entomology, Ceske Budejovice, Czech Republic; 3 Department of Mathematics and Statistics, University of Nevada, Reno, Nevada, United States of America; Sveriges lantbruksuniversitet, SWEDEN

## Abstract

Most of earth’s biodiversity is comprised of interactions among species, yet it is unclear what causes variation in interaction diversity across space and time. We define interaction diversity as the richness and relative abundance of interactions linking species together at scales from localized, measurable webs to entire ecosystems. Large-scale patterns suggest that two basic components of interaction diversity differ substantially and predictably between different ecosystems: overall taxonomic diversity and host specificity of consumers. Understanding how these factors influence interaction diversity, and quantifying the causes and effects of variation in interaction diversity are important goals for community ecology. While previous studies have examined the effects of sampling bias and consumer specialization on determining patterns of ecological networks, these studies were restricted to two trophic levels and did not incorporate realistic variation in species diversity and consumer diet breadth. Here, we developed a food web model to generate tri-trophic ecological networks, and evaluated specific hypotheses about how the diversity of trophic interactions and species diversity are related under different scenarios of species richness, taxonomic abundance, and consumer diet breadth. We investigated the accumulation of species and interactions and found that interactions accumulate more quickly; thus, the accumulation of novel interactions may require less sampling effort than sampling species in order to get reliable estimates of either type of diversity. Mean consumer diet breadth influenced the correlation between species and interaction diversity significantly more than variation in both species richness and taxonomic abundance. However, this effect of diet breadth on interaction diversity is conditional on the number of observed interactions included in the models. The results presented here will help develop realistic predictions of the relationships between consumer diet breadth, interaction diversity, and species diversity within multi-trophic communities, which is critical for the conservation of biodiversity in this period of accelerated global change.

## Introduction

The devaluation of natural history and taxonomy has added to the failure of ecologists to document biodiversity and subsequently to understand the magnitude and consequences of the growing extinctions caused by global change [1]. Knowledge of basic natural history is especially important for quantifying biotic interaction diversity, which encompasses most of earth’s diversity [2], and should be tightly linked to variables such as community stability and ecosystem services [3,4]. The loss of interaction diversity is one of the least understood responses to species extinctions, partly because it has not been consistently treated as a response variable in theoretical or empirical studies of biodiversity and because getting good quantitative data on interaction diversity often requires considerable fieldwork over time. Although network approaches have provided more focus on the structure of species interactions within communities, few analyses are based on detailed natural history data that is linked with experimental evidence of observed interactions actually occurring together (e.g., [5–7]). In contrast, using a standardized sampling approach allows for a more rigorous and repeatable resolution of interaction networks at any appropriate scale [3], but it is not clear how much sampling is necessary for accurate measurements nor how relevant small local interaction networks are to larger scale network properties [8,9].

We define interaction diversity as a measure that combines the relative abundance and richness of interactions linking species together into dynamic biotic communities at multiple scales [3,10–13]. For this metric of diversity, the calculation of richness, diversity indices, and rarefaction diversity is based on experimentally established links between interacting individuals rather than species alone, or alternatively, lists of observations of species found in the same area to determine network nodes and edges. Trophic interactions, such as enemy-herbivore-plant interactions, have large effects on all ecosystem attributes and are well studied [3,14,15], thus tri-trophic webs are suitable systems for examining networks and interaction diversity. Here we focus on this interaction diversity across multiple trophic levels.

Since most communities can never be completely sampled, and the true community values of diversity and other network parameters are impossible to precisely quantify at community scales larger than a hectare, careful sampling approaches are necessary for characterizing interaction diversity [16]. Here we simulate a standardized sampling effort that accumulates individual interactions until each interaction has been accounted for. Utilizing this sampling approach mimics existing systematic sampling protocols in the field, such as standardized plots (e.g., [17]), and allows the comparison of interaction diversity across a broad range of community types. Furthermore, our approach permits us to identify differences between the actual community and a subsample of the community, as certain community characteristics may be more sensitive to disparate sampling efforts than others [9,18,19].

Recently, Fründ et al. [9] investigated the effects of sampling bias on quantifying specialization in bipartite networks and found significant effects of sampling bias on selected properties, while identifying network parameters that are robust to limited sampling. However, this investigation was restricted to two-trophic levels and the range of taxonomic richness and degree of specialization of their simulated communities was narrow. To add to this existing work, we simulated 1000 tri-trophic communities with representative combinations of species richness, taxonomic abundance, and consumer diet-breadth, allowing for a comprehensive investigation into the determinants of interaction diversity across a wide-range of multitrophic communities [3,13,20–25].

The focus of this study was to test specific hypotheses about the relationships between community species diversity, consumer diet breadth, interaction diversity, and network structure. We addressed the following questions with simulation and statistical models:

1. Does interaction diversity asymptote more quickly than species diversity from a discrete sample size or area?
2. What are the interactive effects of consumer diet breadth and species diversity on interaction diversity?
3. Are the combined effects of richness, abundance, and diet breadth on interaction diversity modified by the number of interactions that are observed?

We sampled from simulated networks of interacting trophic levels; mimicking field sampling methods outlined in Dyer et al. [3] and tested relevant paths from a specific structural equation meta model (SEMM, *sensu* [26]) with hypothesized causal relationships between diet breadth and interaction diversity.

## Methods

### Food web simulation

The goal of this model was to generate a random plant-herbivore-parasitoid tri-trophic food web, with interactions only between adjacent trophic levels. Each community is generated to represent the scale of a single study site and are based on several pre-specified properties as inputs to investigate possible contributions to interaction diversity. Specifically, these inputs are the number of species at each trophic level (i.e., richness; *R*_*1*_, *R*_*2*_, *R*_*3*_), the overall abundance of each trophic level (i.e., abundance; *A*_*1*_, *A*_*2*_, *A*_*3*_), and a diet breadth parameter (*α*_*2*_, *α*_*3*_) for the consumers that determines the diet breadth distribution for that trophic level according to a truncated discrete Pareto distribution [17].

The abundance distribution for trophic level *i* was constructed by taking a random sample of size *R*_*i*_ from a lognormal distribution with *μ* = 0 and *σ* = 1, scaled to sum to the prespecified overall abundance *A*_*i*_, and then rounded to the nearest integer [27]. We denoted the abundance of species *j* in trophic level *i* as *A*_*ij*_, where $A_{i}\approx \sum_{j\;=\;1}^{R_{i}}A_{ij}$. Individual diet breadth values (number of species each consumer has in their diet) were assigned to each species to get an empirical distribution that represents the desired discrete truncated Pareto distribution of specialization within the consumer trophic levels. These values were obtained by calculating density values for a (continuous) Pareto I distribution (truncated at the number of species at the lower trophic level) with survival function (aka complementary CDF) *S(y) = (1/y)*^*α*^.

The lists of resource species that each species potentially consumes were then sampled (with replacement) uniformly from the list of species in the lower, adjacent trophic level. In sampling real systems in the field, individual consumers are assumed to have been found by sampling their resource (i.e., herbivores are detected by inspecting host plants, and parasitoids are found by inspecting host herbivores). Therefore we assumed each individual parasitoid/enemy is associated with an individual herbivore, and each individual herbivore with an individual plant. In other words, there is never more than one individual consumer on an individual host, though there are several individuals within a species, so you can have multiple interactions occurring between those two species. Interactions among individuals were therefore constructed as follows. Individual herbivores of species *j* (recall there are *A*_*2j*_ such individuals) were assigned a plant species by cycling through the list of species in their diet. Then each individual plant is assigned an individual herbivore, based on these assignments, and we assume only one herbivore individual per plant individual. This is repeated for each herbivore species until no unoccupied plants remain. Individual herbivores that remained in the community from the original distributions were then removed from the community if all potential host plants are occupied. This process was repeated for enemies, assigning them to herbivores under the same one-to-one assumption, and any unassociated parasitoids are removed from the community. This often resulted in fewer individuals and species, compared to the initial generated values of the communities.

Our randomly assembled food webs were generated by sampling *R*_*i*_ randomly from the set of integers {3, 4,…, 120} and *α*_*i*_ randomly over the interval [1,5]. Total abundances for each trophic level *A*_*i*_ were randomly sampled from the integers {3, 4,…, 500}. These initial values represent the potential values in the realized networks, but will not necessarily match following the sampling procedure. The specific distributions for species richness, relative abundances, and alpha parameters were based on food web data from sites across the Americas [24]. Using this approach, we generated 1000 random food webs.

### Food web sampling

The community was subsampled by randomly selecting individual plants and for each subsample, an individual plant had at most one herbivore and at most one enemy associated with that herbivore. Randomly sampled rows from each local interaction food web were used to calculate the cumulative interaction diversity for each sample. Sampled interaction diversity was calculated using the inverse of the Simpson’s entropy (1/D) for each cumulative plant-herbivore, herbivore-enemy, and plant-herbivore-enemy interaction. Sampling was completed once all plant individuals within each local community were sampled. Each community differed in the number of species, the numbers of individuals within each species, and the diet breadth assigned to each consumer species. Sampling within the local community occurred without replacement. In summary, the assumptions for the simulation were: 1) a lognormal distribution of species abundances for all trophic levels; 2) a truncated discrete Pareto distribution of consumer diet breadths; 3) complete detection of all herbivores and parasitoids associated with an individual plant; 4) only one individual of a consumer species per individual of a resource species.

### Total network analysis

We quantified network-level connectance to identify how species richness and specialization influence the structure of entire networks; connectance is a commonly used network parameter [18,19]. To accomplish this, we assembled three separate, but not mutually exclusive, networks within each individual local community described above. A plant-herbivore (PH), herbivore-enemy (HE), and plant-herbivore-enemy (PHE) network were assembled separately to quantify connectance and compare outcomes when examining two- or three-trophic-level networks.

A weighted network was constructed from each local community by generating a bipartite matrix with the abundance of interactions that occurred between individuals of each community. PH and HE matrices were built based on each local community to calculate network-level properties concerning two trophic levels. To investigate PHE networks, we generated a matrix of producers (e.g. plants and herbivores) and consumers (e.g. herbivores and enemies) and quantified network-level properties similarly to the previously mentioned bipartite networks. For each distinct network (e.g., PH, HE, PHE), the R-package "bipartite" (version 2.05) was utilized to quantify connectance [28]. In all subsequent network analyses, empty columns and rows were deleted before calculating network-level metrics. These values were integrated with other diversity measurements from our sampling scheme to investigate the desired relationships.

### Rarefaction analyses

To compare the accumulation rates of species and interactions in a given local community, we used rarefaction curves and the Chao1 estimator of richness [29]. We generated rarefaction curves using the ‘vegan’ package (version 2.2–1) in R [30] and calculated the slope of each rarefaction curve at the number of samples it took to sample half the total richness for each local community. These values allowed us to compare the accumulation rates between species and interactions across a wide range of local communities. We estimated the richness for interactions and species using the Chao1 non-parametric estimator of richness [29]. Chao1 estimates of richness were calculated for PH, HE, and PHE networks. Specifically, for the PHE networks, only complete PHE interactions were used. Slopes and estimated Chao1 richness were compared using Bayesian estimation for two groups in the R package “BEST” [31,32]. This method provides an alternative to classic t-tests and creates posterior estimates for group means and 95% high-density intervals (HDI). Point estimates and 95% HDI were used to identify differences between sampled interactions and species for all 1000 local communities. The mean and standard deviation of the observed differences between interactions and species networks served as priors. Given the large sample size, the method provides robust posterior probabilities identifying differences between sample means. Differences were considered significant if the 95% HDI did not overlap. All web simulations and network analyses were performed using program R (version 3.3.2) [33].

### Statistical analysis

Linear regression and structural equation models were used to identify the relative effects of taxonomic diversity and diet breadth on interaction diversity and other network structure metrics. We assessed specific path models to test a previously hypothesized structure equation meta-model. Path coefficients for direct effects were obtained from the structural equation model, whereas indirect effects were calculated as the product of direct effects in any given pathway. For our *a priori* specified structural equation model, we identified causal relationships to formulate a simple set of paths with three exogenous variables (plant abundance, herbivore diet breadth, enemy diet breadth) predicting four endogenous variables (interaction diversity, interaction density, species diversity, connectance); no latent variables were used. Specifically, on the basis of literature, our own empirical data, and assumptions of the simulations, all exogenous variables were predicted to increase interaction diversity, species diversity, and connectance. In addition, these exogenous variables were expected to have positive effects on connectance via interaction diversity and density. We tested the fit of this model using SAS (PROC CALIS) and utilized the reticular action model (RAM—a covariance structure model) to specify the models [34]. Starting values for the parameter estimates were determined by using a combination of three methods: observed moments of variables, the McDonald method, and two-stage least squares. The estimation method for the model was maximum likelihood, and the Levenberg-Marquardt algorithm was used to iterate solutions for optimization. The χ^2^ for the absolute index was used to assess the fit of the model, with P > 0.05 (with 2 df) as an indication of a good fit to the data. Residuals met assumptions for multiple regressions. This approach was utilized for the full communities generated by our simulations as well as for random samples from each community that started at 5 interactions sampled up to 500 interactions sampled and path coefficients were compared from the identical models across these sample sizes. Comparing coefficients across a range of sample sizes allowed us to investigate how predicted relationships among variables changes as the number of observed interactions increase, which is analogous to changing the size of the plot or local community.

We also used simple linear regression to examine how consumer diet breadth and taxonomic diversity influence the association between interaction and species diversity. Species diversity was regressed against interaction diversity and the residuals from that model were used as a dependent variable in subsequent linear models. Using residuals as a dependent variable allowed us to identify whether relationships between species and interaction diversity differed under various community conditions, such as specialized or generalized consumers. Linear regressions were performed to identify whether consumer diet breadth, taxonomic abundance, and species richness significantly altered relationships between interaction and species diversity using these residuals. This analysis was implemented for each distinct network (e.g., PH, HE, PHE). The mean observed diet breadth for consumers was utilized as a measure of specialization. Diet breadth was restricted to mean herbivore diet breadth for PH networks, mean enemy diet breadth for HE networks, and the mean diet breadth among herbivores and enemies for PHE networks. The sum of species richness and taxonomic abundance across all trophic levels in the local network was used for measures of richness and abundance. These analyses were performed using program R (version 3.3.2) [33].

## Results

### Interaction and species rarefaction curves

**Fig 1:**
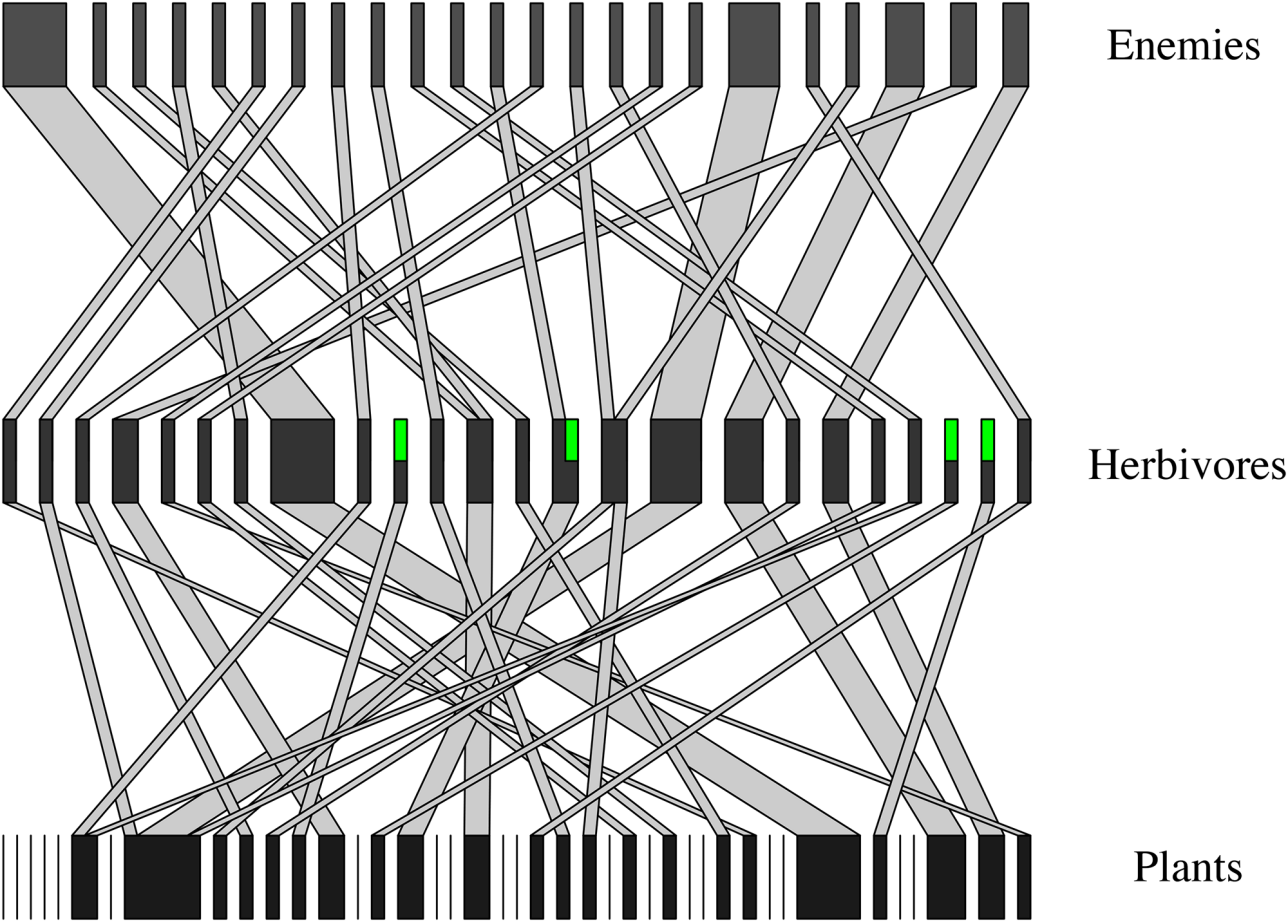
A randomly selected tri-tropic network produced from one of the 1000 simulations. Each black bar is a node representing a unique species, while the grey bars are edges connecting the black bars and represent observed interactions between those two species. Green sections within some of the black bars represent individuals within that particular species that were present in the community, but not involved in trophic interactions (e.g., plants without herbivores). The width of each edge and node within the network denotes the abundance of sampled interactions or species. Only species that were sampled are shown in this network. Numbers above each node denote the species identification number from that particular simulation.

1000 different local communities were generated and cumulatively sampled (see Fig 1 for an example network). Interaction and species rarefaction curves for all PH, HE, and PHE networks yielded variable rarefaction curves among the local communities, between interactions and species, and among the three networks (e.g. PH, HE, PHE) (S1 Fig). Each step in the accumulation curves is analogous to different levels of sampling due to differences in scales (e.g., plots recommended in [3]) or due to error or limited sampling.

**Fig 2:**
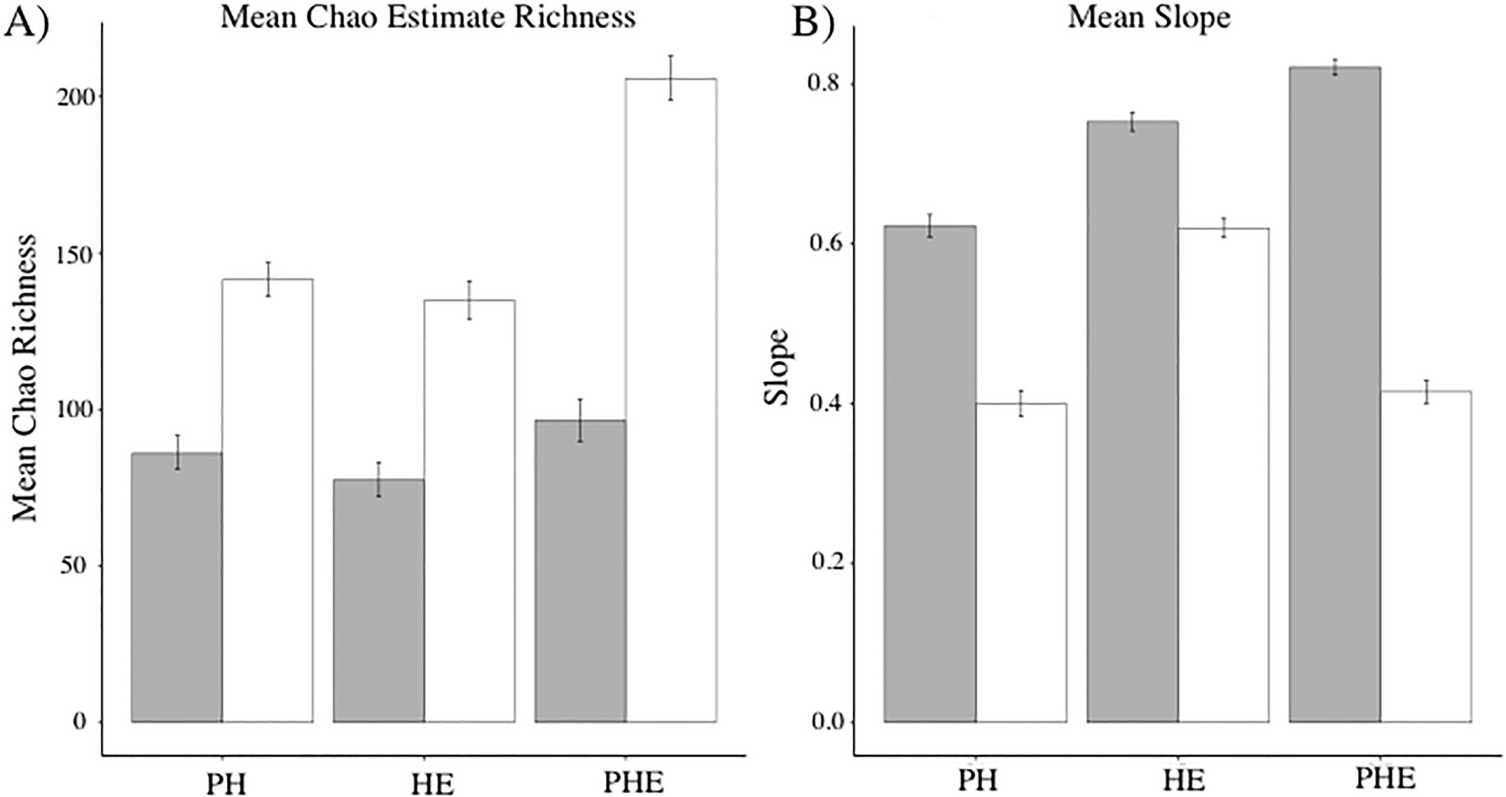
Posterior probabilities of: A) mean Chao1 estimates of richness for species and interactions, and B) the mean slope of rarefaction curves for species and interactions. Interactions are displayed in grey, while species are shown in white. The error bars represent the 95% High Density Intervals (HDI). Mean slopes were acquired by calculating the slope of each rarefaction curve when half of the species or interactions were sampled. Chao1 estimates of richness were acquired using the ‘estimateR’ function in the *vegan* package in R.

The Bayesian estimation of two groups identified significant differences in the mean Chao1 estimator of richness between interactions and species in PH (HDI_sp_ = 136–146, HDI_int_ = 80–91, HDI_diff_ = 48–63), HE (HDI_sp_ = 129–140, HDI_int_ = 72–82, HDI_diff_ = 49–65), and PHE networks (HDI_sp_ = 198–213, HDI_int_ = 89–103, HDI_diff_ = 99–118)(Fig 2A). The effect size was similar in PH (Effect size = 0.95) and HE (Effect size = 0.94) networks and largest for the PHE only network (Effect Size = 1.45). Mean Chao1 estimates of species richness were consistently greater than interactions in all three networks.

Bayesian estimates of the mean slope at the number of samples it took to accumulate half of the total richness (a value analogous to the Michaelis constant in Michaelis-Menton enzyme dynamics) differed significantly among species and interaction rarefaction curves, and among the three network types (Fig 2B)(i.e. PH, HE, PHE). Rarefaction slopes of PH (HDI_sp_ = 0.38–0.42, HDI_int_ = 0.61–0.64, HDI_diff_ = 0.21–0.24), HE (HDI_sp_ = 0.60–0.63, HDI_int_ = 0.74–0.76, HDI_diff_ = 0.12–0.15), and PHE (HDI_sp_ = 0.40–0.43, HDI_int_ = 0.81–0.83, HDI_diff_ = 0.39–0.43) networks consistently higher than species. The estimated difference between species and interactions was greatest in PHE networks. Effect size was smallest when investigating HE (Effect Size = -0.73) networks, but greatest within the PHE networks (Effect Size = -2.73).

### Relationships between species and interaction diversity

**Fig 3:**
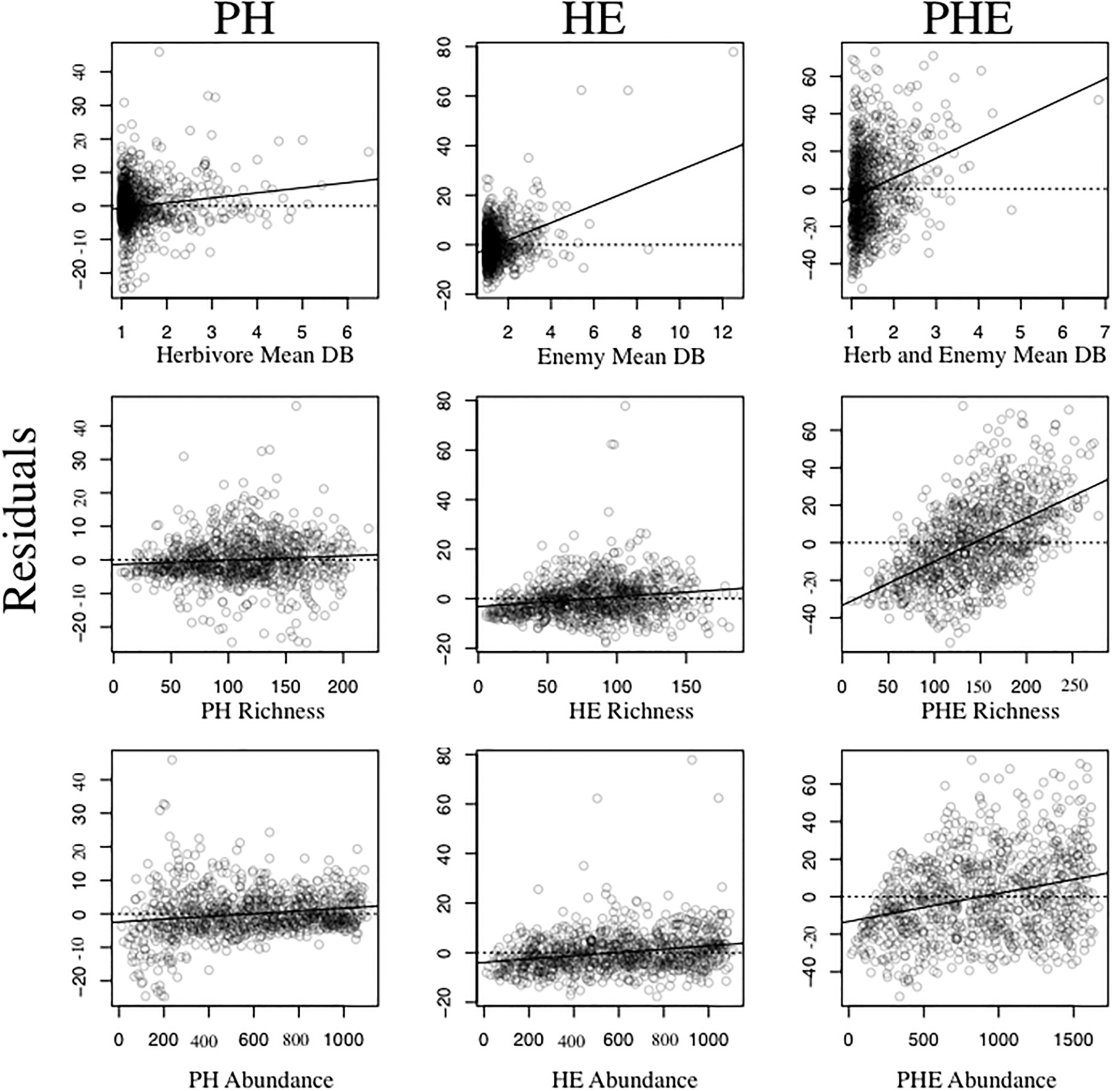
Summary plots of semi-partial correlations between the residuals of species diversity and interaction diversity (these residuals are on the y-axis) and mean consumer diet breadth, species richness, and total abundance (these three parameters are on the x-axis). We investigated this relationship for all three networks (e.g. PH, HE, PHE). The top three panels represent changes in mean diet breadth for each consumer trophic level; mean herbivore and enemy diet breadth were used for the PH and HE networks respectively, while the mean diet breadth for all consumers (herbivores plus enemies) was used for PHE networks. The middle three panels denote community richness for each respective network, which is the total number of species found in all trophic levels. The lower panel displays semi-partial correlations with total community abundance, which equals the sum of all individuals within each trophic level. The solid black lines are least squares regression lines.

The correlation between species and interaction diversity was strongest among PH networks (Pearson’s Corr. = 0.96, p < 0.001) and gradually decreased with HE (Pearson’s Corr. = 0.93, p < 0.001) and PHE networks (Pearson’s Corr. = 0.35, p < 0.001). This pattern remained consistent when the slope and coefficient of determination in linear models was examined (R^2^) (Fig 3; S1 Table).

Diet breadth, species richness, and species abundance all significantly influenced the association between interaction and species diversity (partly due to the high power associated with large sample sizes), but the strength of the effects differed among the networks being investigated (Fig 3; S2 Table). Communities with greater mean consumer diet breadth (i.e. increased generalization) resulted in more positive residuals between species and interaction diversity in PH networks (β = 1.5, P<0.001) (Fig 3). Positive residuals in this case signify higher values of interaction diversity then would be expected given the diversity of species. Similar, but larger effects of diet breadth on relationships between species and interaction diversity were observed in HE (β = 3.53, P < 0.001) and PHE (β = 10.6, P < 0.001) networks (Fig 3).

The effect of species richness on the relationships between species and interaction diversity was significant for all three networks (Fig 3; S2 Table). Increased species richness was positively associated with the residual values of PH (β = 0.013, P = 0.007) and HE networks (β = 0.039, P<0.001). Relationships to PHE network (β = 0.23, P<0.001) residuals displayed the most pronounced, positive linear relationship with increased species richness. These results revealed that local communities with higher values of species richness yielded more interactions than expected based on the number of species present in the community and that effect is strongest when three trophic levels is considered. The variance explained within each model improved in successively higher trophic levels and was greatest when all three trophic levels were incorporated in the models (S2 Table).

Abundance revealed statistically significant linear relationships with residual values from all three networks, but the strength of these associations were relatively weak compared to diet breadth and species richness. Total abundance in PH (β = 0.0041, P<0.001) and HE (β = 0.007, P<0.001) networks displayed the weakest association with residual values (S2 Table). Total abundance within PHE networks revealed the largest positive estimate, but the slope was still noticeably small (β = 0.015, P<0.001). In two of three cases (e.g., richness and abundance), variance explained was greatest when all three trophic levels were considered. Changes in consumer diet breadth resulted in the largest estimate, but models that included richness explained the most variance.

### Path analysis and the effects of sampling

**Fig 4:**
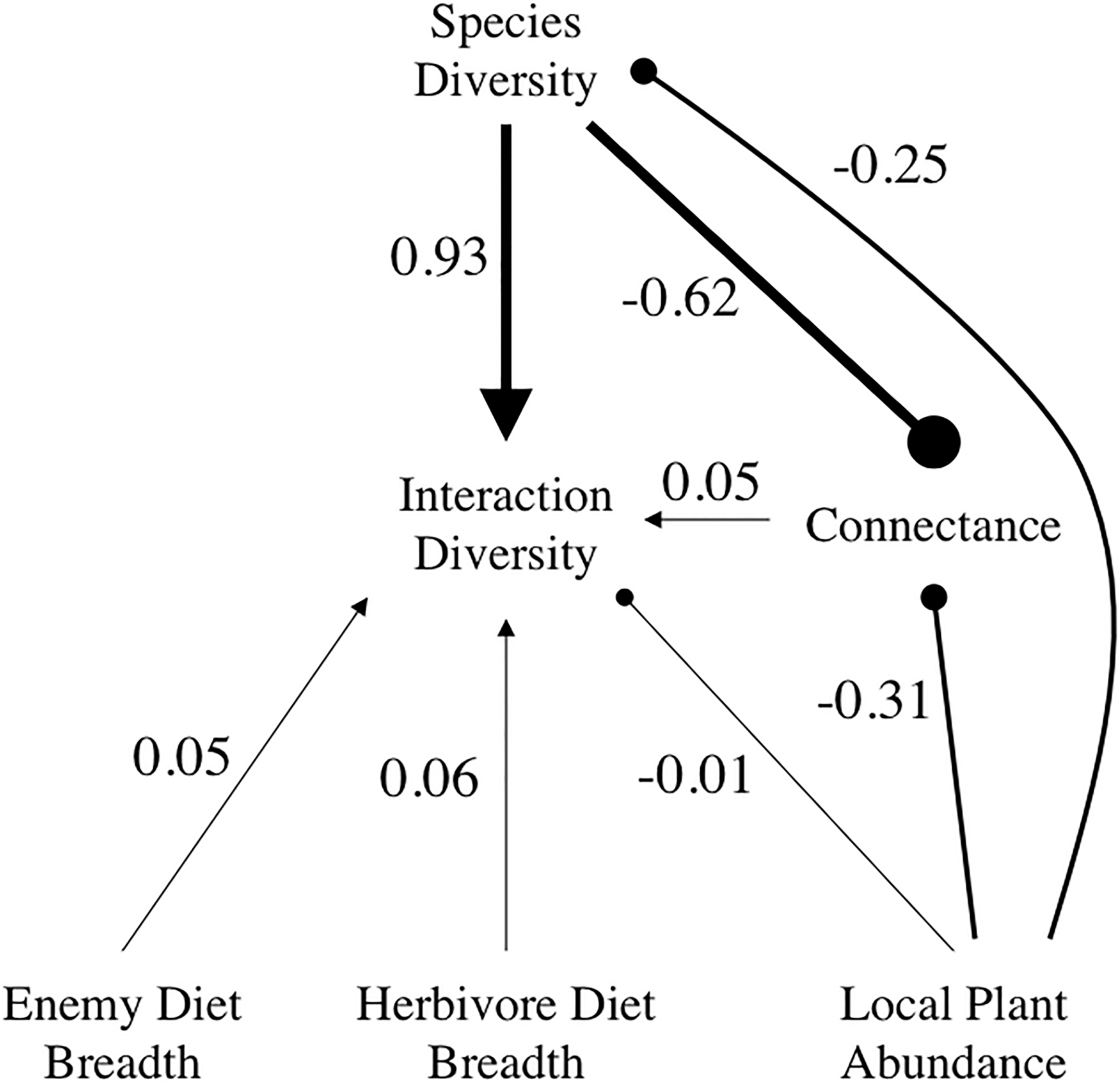
A path diagram summarizing the standardized path coefficients across all 1000 local communities (*χ*^2^ = 3.6, df = 4, P = 0.5; AIC = 36). Each path was chosen based on *a priori* hypotheses, and compared to competing models using AIC and *χ*^2^. Lines ending with an arrow denote positive coefficients, while lines ending with a circle denote negative coefficients. The width of the arrow indicates the relative size of the coefficient.

The best-fit path model, when using all samples, performed significantly better than all other models (Fig 4; *χ*^2^ = 3.6, df = 4, P = 0.5; AIC = 36; delta AIC range = 60–70). Species diversity showed the strongest positive effect on PHE interaction diversity and as predicted, all other variables positively affected interaction diversity (Fig 4). Only total plant abundance within the local community negatively affected, though indirectly, interaction diversity. Thus, communities with more plant individuals had lower values of interaction diversity, but that effect was driven primarily through its strong negative effect on species diversity. The effects of consumer diet breadth and connectance on interaction diversity were both negligible. While species diversity had a strong positive effect on interaction diversity, more species-diverse communities had lower levels of connectance. Local plant abundance within communities had strong negative effects on species diversity and connectance, but weak direct effects on interaction diversity.

**Fig 5:**
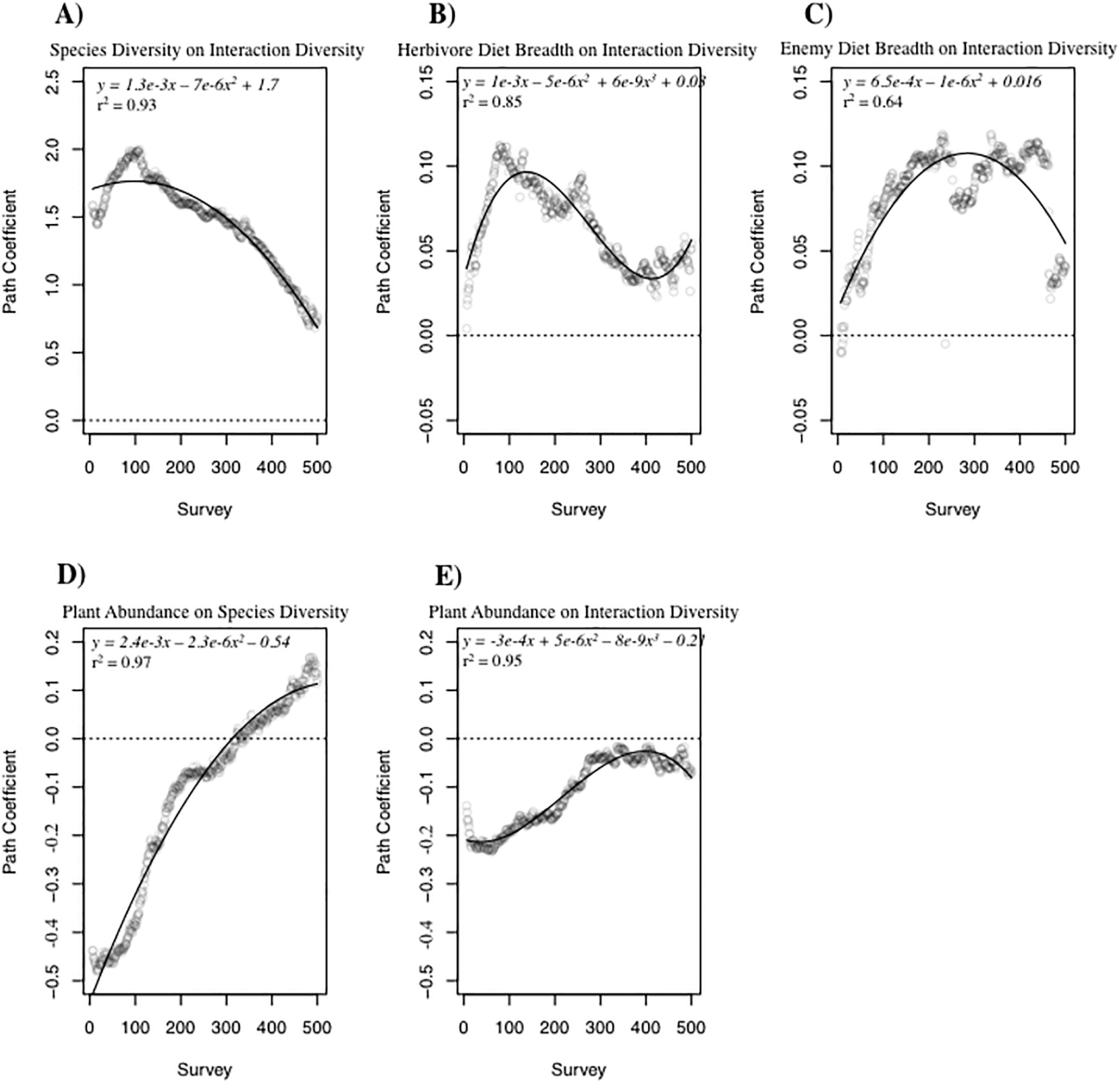
Scatterplots displaying the relationship between the strength of each path coefficient and the number of sampled interactions included in the path analysis (Fig 5), with the exception of paths associated with connectance. The strength of the path coefficient is shown on the y-axis and number of observed interactions included in the model is shown on the x-axis. The solid line represents outcome of linear or polynomial regressions. Path coefficients used in these analyses were significant (P < 0.05).

To understand the sensitivity of each path coefficient to the number of observations included in the path analysis, path coefficients were derived from SEMs that used random samples from each simulated community that started at 5 interactions and increased up to 500 interactions (Fig 5). Due to issues with generating balanced samples for SEM, connectance was not included in this model and therefore the structure of the path model differed from that shown in Fig 4. We consider the random samples to be analogous either to actual sampling in a biotic community or to smaller scale communities that are derived from a regional pool of species and potential interactions.

As the number of observed interactions included in each SEM increased, the strength of each path coefficient varied considerably (Fig 5). The degree to which each path coefficient changed differed among the coefficients and all responded in a non-linear fashion. The direct positive effect of species diversity on interaction diversity decreased significantly as more observations were included in the path analysis (Fig 2A). Mean herbivore diet breadth maintained a positive effect on interaction diversity across all observed interactions, but its effect was greatest at intermediate values and decreased as the number of observations exceeded 100 (Fig 2B). The direct effect of enemy diet breadth on interaction diversity was strongest at intermediate (~200–300) numbers of observed interactions (Fig 2C), while the effect of local plant abundance on species diversity increased consistently across sampled interactions (Fig 2D). The effect of local plant abundance ultimately yielded a positive influence on species diversity, and this difference from the model with full webs (Fig 4) was most likely due to the absence of connectance from the path analysis. The effect of plant abundance on interaction diversity also increased as the number of observations increased, but never resulted in a positive effect (Fig 2E).

## Discussion

The interest in interaction diversity as a metric of biodiversity has developed separately from natural history studies that attempt to rigorously document interactions at local and regional scales [25]. Interaction diversity and other network parameters, such as connectance, have been gleaned from loosely constructed networks (e.g., from literature searches or brief observational studies), and these parameters have been utilized as measures relevant to network structure and resilience. But these networks are not realistic since local networks do not include all possible edges among nodes that are present [8]. One reason to examine how relationships among node and edge diversity and network parameters can change with sampling effort or area sampled is to assess the relevance of network analyses based on these putatively empirical regional networks [35]. Such scaling and sampling issues cannot be ignored when this regional network view of interaction diversity is utilized to assess issues associated with relationships between biodiversity, productivity, ecosystem function, and extinction.

Our food web simulation generates hypotheses relevant to the power of sampling actual interactions and calculating the diversity of interacting individuals across a variety of ecological communities. The clearest patterns that emerged and are worth pursuing with empirical data were: 1) randomly assembled networks produce accumulation curves for interaction diversity that reach an apparent asymptote more quickly than species diversity, so interaction diversity may be more practical to estimate than species diversity in real ecosystems—this is especially true at intermediate sample sizes (or local community sizes [3]), where local species diversity is the best predictor of local interaction diversity at multiple sampling scales; 3) consumer diet breadth, defined by a truncated Pareto distribution, may disrupt the strong relationship between interaction and species diversity, as more generalized communities will have higher interaction diversity; 4) species diversity and local plant abundance are also likely to predict other tri-trophic network parameters, such as connectance; and 5) local network parameters are likely to be quite different from the regional networks, and this relationship changes as the networks grow in size.

### The interaction diversity model

Our approach to simulating tri-trophic networks provides randomly assembled quantitative communities that can be separated into discrete bipartite networks nested within a randomly assembled community. This provides an opportunity to investigate how the number and position of trophic interactions influences network-level properties from a discrete sampling procedure [36]. Furthermore, the design of the simulation model provides generous flexibility allowing researchers to modify foundational building blocks of ecological communities, including distributions of consumer diet breadths. Finally, it provides insight into how sample size or spatial scale can affect network properties. The addition of a third trophic level separates our approach from previous simulated network data (e.g., [9,19,37,38], and for both modeling and empirical approaches to ecological networks, expanding to more complex interaction networks should be a focus as it can provide additional information on network dynamics and function. Our model showed that the number and position of trophic levels that are being analyzed, especially when considering plant, herbivore, and natural enemy communities, influence network-level properties. Samples from studies that incorporate higher trophic levels are completely dependent on the successful sampling of associated hosts. This can have significant impacts on the observed structure and diversity of a sampled network. As more trophic levels are included in a network, the dependencies of sampled (or included) interactions increase, which exacerbates problems with large regional networks that actually do not exist locally.

The simulation of tri-trophic networks developed “complete” networks that were assembled with only one assumption—networks consisted of consumers with restricted diets and included realistic numbers of species and interactions (based on empirical interaction diversity data). Our goal was to generate a network that is more consistent with standard neutral assumptions (no assembly rules) combined with niche-based assumptions (specialization), rather than following an abundance-based simulation null model [18]. In the future, the flexibility of our simulation model, which allows the manipulation of richness, abundance, and diet breadth for each trophic level included in the community, will incorporate other assumptions, such as assembly rules [39,40], or to omit the assumption of restricted consumer diet. However, our utilization of a truncated Pareto distribution for host range is well supported in plant-arthropod networks [17] and provides a realistic measure of host specialization in multi-trophic networks that include plants, insect herbivores, and parasitoid natural enemies. The manipulation of richness, abundance, and diet breadth and their distributions, allows for a useful tool to compare observed data to simulated data from the model. This can help with determining the importance of diet breadth distribution or degree of specialization versus other factors in sampled networks when exploring relationships between diversity, network processes, and network patterns [9,41,42]. Finally, the subsampling approach can generate smaller networks that are a more realistic representation of interacting species in local food webs [43].

### Species and interaction rarefaction curves

Few studies have attempted to compare rarefaction curves for species and interactions across a wide range of multitrophic communities (but see [44–46]. Rarefaction is used to easily compare measures of richness between communities in which the sampling effort is different and can be useful to help identify the completeness of sampling that has occurred in a community [47]. It is assumed, though never tested, that given the substantially more potential interactions than species, interactions should accumulate much more slowly than species when sampling from a discrete sample area. However, many interactions never occur (i.e. they are forbidden or never observed) and it is possible that interactions are characterized by a more kurtotic distribution than species, which should result in interactions obtaining an apparent asymptote more quickly than species [3,48]. In other words, similar to species distributions, interactions are typically dominated by a few, abundant connections, with many singleton or rare interactions. Therefore, the shape of rarefaction curves may be highly influenced by the abundance distributions, taxonomic richness, and host range of consumers in multi-trophic communities.

The assumptions of our model and the focus on more specialized consumers clearly impose some limits to the generality of our results. For example, the values of interaction richness yielded by this simulation may be considerably lower than species richness due to our high levels of host specialization. A truncated Pareto distribution involves few generalist and many specialist species, and this increase in more limited trophic interactions reduces the number of unique interactions that occur when there are no assembly rules or differences in densities for consumers of different diet breadths. On the other hand, if specialists are always more abundant than generalists, this distribution can increase the number of unique interactions locally. Other networks (e.g., plant-pollinator) have revealed higher numbers of interactions than species (e.g. plants and pollinators) [49–51], but these mutualistic communities are normally characterized by more generalized interactions, have often been regional networks (i.e. large scale), and the networks are almost always based on all visitors rather than true pollinators [52–55]. Furthermore, these communities ignore more subtle factors that affect network parameters and specialization, such as adaptive foraging [54]. The model’s generality may be reduced in other ways, but using a truncated Pareto distribution of host specialization may be the best approach to studying antagonistic interactions, especially those involving plants, insects, and parasitoid natural enemies [24]. However, the simulation approach is adaptable and any distribution of host utilization is possible, and modified assumptions would be necessary for communities other than plant, insect herbivore, and parasitoid communities.

### Associations between species diversity and interaction diversity

As expected, we observed a strong positive correlation between species and interaction diversity, but this relationship was more stable than anticipated across the diverse range of communities, scales, and sample sizes. We hypothesized that consumer diet breadth and other community parameters (e.g. richness and abundance) should have altered the correlation between interaction and species diversity more than what we observed. Specifically, more specialized communities (higher α-parameters) result in lower positive correlation coefficients (fewer links per node) due to the decrease of interactions that involve generalist species. Based on our simulations, notable changes in the correlation coefficient or slope among species and interaction diversity across a wide range of combinations of community parameters were observed (Fig 3, S1 and S2 Tables), but the effects were weaker than expected. More specialized communities displayed more negative residuals, which suggests that there are fewer interactions than expected based on the number of species present in the community. Although this effect was small, it supports the hypothesis that generalized interactions are rare, but have large effects on interaction diversity locally [3]. Generally, community parameters (e.g., richness, abundance, diet breadth) had little effect on the relationship between species and interaction diversity, probably due to the lack of assembly rules and low numbers of generalists. The main parameters that altered the associations between species and interaction diversity were the number of trophic levels.

An important contribution of our simulation is that it included more than two trophic levels in an effort to understand how the position and number of trophic levels in a community can drive relationships between species and interaction diversity. Many network studies have been limited to plant-pollinator or plant-herbivore networks, yet communities are far more complex, and patterns of interaction diversity and network topology from two-trophic-level analyses are likely different from more realistic multi-trophic communities. Our results revealed that when incorporating three trophic levels, the community parameters (e.g., diet, richness, and abundance) all have stronger impacts on the relationship between species and interaction diversity. This is likely due to the contingent nature of sampling partners at lower trophic levels to acquire individuals at higher trophic levels. In other words, the likelihood of sampling enemies is founded on the likelihood of sampling an herbivore, which results in a propagation of effects, changing the probability density functions of interactions differently from species density functions.

While this is an unavoidable sampling artifact, it is important to consider when drawing conclusions about the observed structure of a multi-trophic ecological network. Thus, when investigating more than two trophic levels, the impacts of consumer specialization and species richness are magnified in driving food web patterns and decrease associations between species and interaction diversity [56]. Utilizing interaction diversity, as a metric of biodiversity, to help with conservation and management issues will be most useful when more than two trophic levels are investigated. Otherwise, species diversity should be a reasonable proxy for interaction diversity when a community is dominated by only plants and herbivores since disparities between interaction and species diversity are lowest for two trophic levels.

### Effects of primary productivity, diet breadth, species diversity, and number of observed interactions on network structure

We observed considerable variance in interaction diversity in the assembly of 1000 tri-trophic communities, with the only constraint on consumer diet-breadth distributions. This variance was due to both random effects and partly due to the deterministic effects of the manipulated parameters. By utilizing a path analysis framework we were able to identify direct and indirect effects of multiple community parameters on interaction diversity. Under this framework, species diversity, and to a lesser extent consumer diet breadth revealed the strongest direct effects determining interaction diversity. As expected, species diversity had a strong positive effect on interaction diversity. The effect of herbivore and enemy diet breadth were similarly positive but not very strong. These results are not what we originally predicted given that we expected interaction diversity to be an emergent consequence of distributions of consumer specialization and taxonomic richness [57]. As stated previously, this weak effect of diet breadth was likely due to the highly skewed truncated Pareto distribution.

The relationship between connectance and interaction diversity was relatively weak and shows dissimilar relationships with other variables in the path analysis. This result suggests that connectance and interaction diversity are measuring different qualities of ecological communities and are determined by different factors within a community. Further, if the goal is to conserve biodiversity within a community, connectance does not appear to be a good predictor of diversity of interactions and is negatively related to species diversity [57,58]. However, connectance facilitated both the indirect effects of species diversity and local plant abundance on interaction diversity.

Using a similar path analysis (i.e. without connectance), we found that the number of observations included in the model biases the strength of all path coefficients, and this could also be viewed as a scaling issue—lower numbers of observations in our model are analogous to more localized assemblages within a community. Studies investigating these sampling or scaling effects on ecological network parameters are rare, but they are important because ecological networks are especially vulnerable to sampling effects as well as scale [9,18,59]. One of the more interesting patterns was the sharp decline in the effect of species diversity on interaction diversity as the number of observed interactions increased. This suggests that the effect of species diversity on interaction diversity can be overestimated when the number of observations is insufficient, and that when sufficient observations accumulate the relationship between species and interaction diversity becomes weaker. Similar to what previous studies have found, diet breadth of both herbivores and predators was sensitive to sampling bias [9]. However, identifying the effects of consumer specialization on interaction diversity may be difficult given the nonlinear relationship with the sample size or changes in scale.

## Conclusions

While this model will be useful for developing basic hypotheses concerning the drivers of trophic interaction diversity, there are details in our model that merit further work. We utilized this simulation to test hypotheses about accumulation patterns of species and interactions, but this modeling approach is also appropriate for investigating spatial scaling of interactions and species. A great deal of progress has been made towards understanding species diversity, but we lack even a rudimentary understanding of the determinants and spatial or temporal dynamics of interaction diversity. Food web simulations may be particularly useful to investigate more about the relationships between local and regional interaction diversity [60,61], which will provide insight into the utility of the preponderance of large regional networks that are used to address big issues in ecology and conservation.

In conclusion, we demonstrated that in highly specialized communities, trophic interactions accumulate more quickly than species. We showed that diet breadth and taxonomic richness both interact to influence relationships between species and interaction diversity. Importantly, this model demonstrated that the position and number of trophic levels being investigated strongly impacted correlations among species and interaction diversity, which is critical for biodiversity research and conservation efforts. Interaction and species diversity are most divergent when incorporating more than two trophic levels, so utilizing interaction diversity as a metric of biodiversity will be useful for multi-trophic investigations for both applied and basic research questions such as spatiotemporal dynamics, a biogeographical theory of species interactions [8], and the effects of climate change on biological networks.

## Supporting information

S1 Table Direct relationship between species and interaction diversity as estimated by correlation coefficients, and the beta coefficients from linear regressions between species and interaction diversity. (PDF)

S2 Table Beta coefficient and R^2^ for linear regression of residuals from linear regression between species and interaction diversity and the variable of interest (diet breadth, species richness, abundance). (PDF)

S1 Fig Rarefaction curves for interactions and species from 1000 simulated communities. Rarefaction curves were generated using a modified version of the ‘rarecurve’ function in the R-package, *vegan*. This modification permitted sampling of species and interactions within each community with replacement 500 times. Rarefaction curves were generated for all three networks within each community: Plant-Herbivore (PH), Herbivore-Enemy (HE), and Plant-Herbivore-Enemy (PHE). PHE networks include each unique PHE interaction, excluding PH interactions that were not involved in a HE interaction.
(PDF)

S1 File R-Code to generate tri-trophic networks used in this analysis. (R)

S2 File Raw accumulation data from 1000 model simulations used in this manuscript. (ZIP)

S3 File Analysis and descriptive statistics for each complete tri-trophic network. No accumulation data is included, only the final, observed network.
(ZIP)

## References

[1] Tewksbury JJ, Anderson JG, Bakker JD, Billo TJ, Dunwiddie PW, Groom MJ, et al Natural history’s place in science and society. BioScience. 2014;64: 300–310.

[2] Ohgushi T, Craig TP, Price PW. Ecological communities: plant mediation in indirect interaction webs. Cambridge University Press; 2007.

[3] Dyer LA, Walla TR, Greeney HF, Stireman JO III, Hazen RF. Diversity of Interactions: A Metric for Studies of Biodiversity. Biotropica. 2010;42: 281–289. doi: 10.1111/j.1744-7429.2009.00624.x

[4] Mougi A, Kondoh M. Diversity of interaction types and ecological community stability. Science. 2012;337: 349–351. doi: 10.1126/science.1220529 22822151 10.1126/science.1220529

[5] Novotny V, Basset Y, Miller SE, Weiblen GD, Bremer B, Cizek L, et al Low host specificity of herbivorous insects in a tropical forest. Nature. 2002;416: 841–844. doi: 10.1038/416841a 11976681 10.1038/416841a

[6] Janzen DH, Hajibabaei M, Burns JM, Hallwachs W, Remigio E, Hebert PDN. Wedding biodiversity inventory of a large and complex Lepidoptera fauna with DNA barcoding. Philosophical Transactions of the Royal Society B: Biological Sciences. 2005;360: 1835–1845. doi: 10.1098/rstb.2005.1715 16214742 10.1098/rstb.2005.1715 PMC1609230

[7] Ballantyne G, Baldock KC, Willmer PG. Constructing more informative plant–pollinator networks: visitation and pollen deposition networks in a heathland plant community. Philosophical Transactions of the Royal Society B: Biological Sciences. 2015;282: 20151130. 10.1098/rspb.2015.1130 PMC4571695 26336181

[8] Poisot T, Canard E, Mouillot D, Mouquet N, Gravel D, Jordan F. The dissimilarity of species interaction networks. Ecology Letters. 2012;15: 1353–61. doi: 10.1111/ele.12002 22994257 10.1111/ele.12002

[9] Fründ J, McCann KS, Williams NM. Sampling bias is a challenge for quantifying specialization and network structure: lessons from a quantitative niche model. Oikos. 2016;125: 502–513. doi: 10.1111/oik.02256

[10] Janzen DH. The deflowering of Central America. La deforestación de Centroamérica. Natural History. 1974;83.

[11] Thompson JN. Evolutionary ecology and the conservation of biodiversity. Trends in Ecology and Evolution. 1996;11: 300–303. 21237854 10.1016/0169-5347(96)20048-5

[12] Thompson JN. Conserving interaction biodiversity The Ecological Basis of Conservation. Springer; 1997 pp. 285–293.

[13] Dáttilo W, Dyer L. Canopy openness enhances diversity of ant–plant interactions in the Brazilian Amazon rain forest. Biotropica. 2014;46: 712–719.

[14] Gross T, Rudolf L, Levin SA, Dieckmann U. Generalized models reveal stabilizing factors in food webs. Science. 2009;325: 747–750. doi: 10.1126/science.1173536 19661430 10.1126/science.1173536

[15] Jiang L, Joshi H, Patel SN. Predation alters relationships between biodiversity and temporal stability. The American Naturalist. 2009;173: 389–399. doi: 10.1086/596540 19199526 10.1086/596540

[16] Novotny V, Miller SE, Baje L, Balagawi S, Basset Y, Cizek L, et al Guild-specific patterns of species richness and host specialization in plant-herbivore food webs from a tropical forest. Journal of Animal Ecology. 2010;79: 1193–1203. doi: 10.1111/j.1365-2656.2010.01728.x 20673235 10.1111/j.1365-2656.2010.01728.x

[17] Forister ML, Novotny V, Panorska AK, Baje L, Basset Y, Butterill PT, et al The global distribution of diet breadth in insect herbivores. Proceedings of the National Academy of Sciences. 2015;112: 442–447. doi: 10.1073/pnas.1423042112 25548168 10.1073/pnas.1423042112 PMC4299246

[18] Dormann C, Fründ J, Blüthgen N, Gruber B. Indices, graphs and null models: analyzing bipartite ecological networks. The Open Ecology Journal. 2009;2: 7–24.

[19] Thébault E, Fontaine C. Stability of ecological communities and the architecture of mutualistic and trophic networks. Science. 2010;329: 853–856. doi: 10.1126/science.1188321 20705861 10.1126/science.1188321

[20] Darwin C. On the origin of species by means of natural selection, or, the preservation of favoured races in the struggle for life. 15th ed London: J. Murray; 1859. PMC5184128 30164232

[21] Wallace AR. Tropical nature, and other essays. Macmillan and Company; 1878.

[22] Novotny V, Drozd P, Miller SE, Kulfan M, Janda M, Basset Y, et al Why are there so many species of herbivorous insects in tropical rainforests? Science. 2006;313: 1115–1118. doi: 10.1126/science.1129237 16840659 10.1126/science.1129237

[23] Novotny V, Miller SE, Hulcr J, Drew RAI, Basset Y, Janda M, et al Low beta diversity of herbivorous insects in tropical forests. Nature. 2007;448: 692 doi: 10.1038/nature06021 17687324 10.1038/nature06021

[24] Dyer LA, Singer MS, Lill JT, Stireman JO, Gentry GL, Marquis RJ, et al Host specificity of Lepidoptera in tropical and temperate forests. Nature. 2007;448: 696 doi: 10.1038/nature05884 17687325 10.1038/nature05884

[25] Dyer L, Wagner D, Greeney H, Smilanich AM, Massad T, Robinson M, et al Novel Insights into Tritrophic Interaction Diversity and Chemical Ecology Using 16 Years of Volunteer-Supported Research. American Entomologist. 2012;58: 15–19.

[26] Jiménez-Alfaro B, Chytrỳ M, Mucina L, Grace JB, Rejmánek M. Disentangling vegetation diversity from climate–energy and habitat heterogeneity for explaining animal geographic patterns Ecology and evolution. 2016; 10.1002/ece3.1972 PMC4747316 26900451

[27] Magurran AE. Measuring biological diversity. John Wiley & Sons; 2013.

[28] Dormann CF, Gruber B, Fründ J. Introducing the bipartite package: analysing ecological networks. interaction. 2008;1: 0–2413793.

[29] Chao A. Nonparametric estimation of the number of classes in a population. Scandinavian Journal of Statistics. 1984; 265–270.

[30] Oksanen J, Blanchet FG, Kindt R, Legendre P, Minchin PR, O’Hara RB, et al. vegan: Community Ecology Package, R Package version 2.2–1. http://CRAN.R-project.org/package=vegan; 2015.

[31] Kruschke JK. Bayesian estimation supersedes the t test. Journal of Experimental Psychology: General. 2013;142: 573. 22774788 10.1037/a0029146

[32] Kruschke J, Meredith M. BEST: Bayesian Estimation Supersedes the t-Test. R Package version 0.5.0 [Internet]. 2015. http://cran.cnr.berkeley.edu/web/packages/BEST/vignettes/BEST.pdf

[33] R Core Team. R: A Language and Environment for Statistical Computing, version 3.3.2 [Internet]. Vienna, Austria: R Foundation for Statistical Computing; 2014 http://www.R-project.org

[34] SAS Institute Inc. Base SAS^®^ 9.3 Procedures Guide. Cary, NC: SAS Institute Inc.; 2011.

[35] Schleuning M, Fründ J, Klein A-M, Abrahamczyk S, Alarcón R, Albrecht M, et al Specialization of mutualistic interaction networks decreases toward tropical latitudes. Current biology. 2012;22: 1925–1931. doi: 10.1016/j.cub.2012.08.015 22981771 10.1016/j.cub.2012.08.015

[36] Jordán F, Osváth G. The sensitivity of food web topology to temporal data aggregation. Ecological Modelling. 2009;220: 3141–3146.

[37] Blüthgen N. Why network analysis is often disconnected from community ecology: a critique and an ecologist’s guide. Basic and Applied Ecology. 2010;11: 185–195.

[38] Tylianakis JM, Laliberte E, Nielsen A, Bascompte J. Conservation of species interaction networks. Biological Conservation. 2010;143: 2270–2279. doi: 10.1016/j.biocon.2009.12.004

[39] Keddy PA. Assembly and response rules: two goals for predictive community ecology. Journal of Vegetation Science. 1992;3: 157–164.

[40] Weiher E, Keddy P. Ecological assembly rules: perspectives, advances, retreats. Cambridge University Press; 2001.

[41] Staniczenko PP, Kopp JC, Allesina S. The ghost of nestedness in ecological networks. Nature Communications. 2013;4: 1391 doi: 10.1038/ncomms2422 23340431 10.1038/ncomms2422

[42] Peralta G, Frost CM, Rand TA, Didham RK, Tylianakis JM. Complementarity and redundancy of interactions enhance attack rates and spatial stability in host–parasitoid food webs. Ecology. 2014;95: 1888–1896. 25163121 10.1890/13-1569.1

[43] Staniczenko PP, Lewis OT, Tylianakis JM, Albrecht M, Coudrain V, Klein A-M, et al Predicting the effect of habitat modification on networks of interacting species. Nature Communications. 2017;8: 792 doi: 10.1038/s41467-017-00913-w 28986532 10.1038/s41467-017-00913-w PMC5630616

[44] Vazquez DP, Chacoff NP, Cagnolo L. Evaluating multiple determinants of the structure of plant–animal mutualistic networks. Ecology. 2009;90: 2039–2046. 19739366 10.1890/08-1837.1

[45] Burkle LA, Knight TM. Shifts in pollinator composition and behavior cause slow interaction accumulation with area in plant–pollinator networks. Ecology. 2012;93: 2329–2335. 23236904 10.1890/12-0367.1

[46] López-Carretero A, Díaz-Castelazo C, Boege K, Rico-Gray V. Evaluating the spatio-temporal factors that structure network parameters of plant-herbivore interactions. PloS ONE. 2014;9: e110430 doi: 10.1371/journal.pone.0110430 25340790 10.1371/journal.pone.0110430 PMC4207832

[47] Gotelli NJ, Colwell RK. Quantifying biodiversity: Procedures and pitfalls in the measurement and comparison of species richness. Ecology Letters. 2001;4: 379–391.

[48] González-Varo JP, Traveset A. The labile limits of forbidden interactions. Trends in Ecology & Evolution. 2016;31: 700–710. 27471077 10.1016/j.tree.2016.06.009

[49] Gibson RH, Knott B, Eberlein T, Memmott J. Sampling method influences the structure of plant–pollinator networks. Oikos. 2011;120: 822–831.

[50] Chacoff NP, Vazquez DP, Lomascolo SB, Stevani EL, Dorado J, Padron B. Evaluating sampling completeness in a desert plant–pollinator network. Journal of Animal Ecology. 2012;81: 190–200. doi: 10.1111/j.1365-2656.2011.01883.x 21815890 10.1111/j.1365-2656.2011.01883.x

[51] Fang Q, Huang S-Q. Plant-pollinator interactions in a biodiverse meadow are rather stable and tight for three consecutive years. Integrative zoology. 2016;11: 199–206. doi: 10.1111/1749-4877.12190 26846890 10.1111/1749-4877.12190

[52] Vázquez DP, Aizen MA. Asymmetric specialization: a pervasive feature of plant-pollinator interactions. Ecology. 2004;85: 1251–1257.

[53] Petanidou T, Kallimanis AS, Tzanopoulos J, Sgardelis SP, Pantis JD. Long-term observation of a pollination network: fluctuation in species and interactions, relative invariance of network structure and implications for estimates of specialization. Ecology Letters. 2008;11: 564–575. doi: 10.1111/j.1461-0248.2008.01170.x 18363716 10.1111/j.1461-0248.2008.01170.x

[54] King C, Ballantyne G, Willmer PG. Why flower visitation is a poor proxy for pollination: measuring single-visit pollen deposition, with implications for pollination networks and conservation. Methods in Ecology and Evolution. 2013;4: 811–818.

[55] Valdovinos FS, Brosi BJ, Briggs HM, Moisset de Espanés P, Ramos-Jiliberto R, Martinez ND. Niche partitioning due to adaptive foraging reverses effects of nestedness and connectance on pollination network stability. Ecology Letters. 2016;19: 1277–1286. doi: 10.1111/ele.12664 27600659 10.1111/ele.12664

[56] Beaver RA. Geographical variation in food web structure in Nepenthes pitcher plants. Ecological Entomology. 1985;10: 241–248.

[57] Beckerman AP, Petchey OL, Warren PH. Foraging biology predicts food web complexity. Proceedings of the National Academy of Sciences. 2006;103: 13745–13749. doi: 10.1073/pnas.0603039103 16954193 10.1073/pnas.0603039103 PMC1560085

[58] Winemiller KO. Must connectance decrease with species richness? The American Naturalist. 1989;134: 960–968.

[59] Nielsen A, Bascompte J. Ecological networks, nestedness and sampling effort. Journal of Ecology. 2007;95: 1134–1141.

[60] Cornell HV, Lawton JH. Species interactions, local and regional processes, and limits to the richness of ecological communities: a theoretical perspective. Journal of Animal Ecology. 1992; 1–12.

[61] Ricklefs R, Schluter D. Species diversity in ecological communities: historical and geographical perspectives. 1993.

