# Supplementary material for: Simulated tri-trophic networks reveal complex relationships between species diversity and interaction diversity
Source: PLoS One. 2018 Mar 26;13(3):e0193822. doi: 10.1371/journal.pone.0193822 (PMC5868776; doi:10.1371/journal.pone.0193822)
Supplement: S1 Table — (PDF) [file pone.0193822.s001.pdf]

S1 Table: Direct relationship between species and interaction diversity as estimated by correlation coefficients, and the beta coefficients from linear regressions between species and interaction diversity

Network	Pearson's Correlation	Beta	R^2
PH	0.97	0.72	0.92
HE	0.94	0.66	0.87
PHE	0.35	0.21	0.12
