# Supplementary material for: Simulated tri-trophic networks reveal complex relationships between species diversity and interaction diversity
Source: PLoS One. 2018 Mar 26;13(3):e0193822. doi: 10.1371/journal.pone.0193822 (PMC5868776; doi:10.1371/journal.pone.0193822)
Supplement: S2 Table — (PDF) [file pone.0193822.s002.pdf]

S2 Table: Beta coefficient and R^2 for linear regression of residuals from linear regression between species and interaction diversity and the variable of interest (diet breadth, species richness, abundance).

Network	Beta Diet Breadth	R^2 Diet Breadth	Beta Richness	R^2 Richness	Beta Abundance	R^2 Abundance
PH	1.5	0.02	0.013	0.006	0.004	0.03
HE	3.5	0.14	0.039	0.03	0.007	0.06
PHE	10.6	0.07	0.230	0.31	0.015	0.09
