# Supplementary material for: Simulated tri-trophic networks reveal complex relationships between species diversity and interaction diversity
Source: PLoS One. 2018 Mar 26;13(3):e0193822. doi: 10.1371/journal.pone.0193822 (PMC5868776; doi:10.1371/journal.pone.0193822)
Supplement: S1 Fig — Rarefaction curves were generated using a modified version of the ‘rarecurve’ function in the R-package, vegan. This modification permitted sampling of species and interactions within each community with replacement 500 times. Rarefaction curves were generated for all three networks within each community: Plant-Herbivore (PH), Herbivore-Enemy (HE), and Plant-Herbivore-Enemy (PHE). PHE networks include each unique PHE interaction, excluding PH interactions that were not involved in a HE interaction. (PDF) [file pone.0193822.s003.pdf]

S1 Fig. **Rarefaction curves for interactions and species from 1000 simulated communities.** Rarefaction curves were generated using a modified version of the 'rarecurve' function in the R-package, *vegan*. This modification permitted sampling of species and interactions within each community with replacement 500 times. Rarefaction curves were generated for all three networks within each community: Plant-Herbivore (PH), Herbivore-Enemy (HE), and Plant-Herbivore-Enemy (PHE). PHE networks include each unique PHE interaction, excluding PH interactions that were not involved in a HE interaction.

S1 Fig.

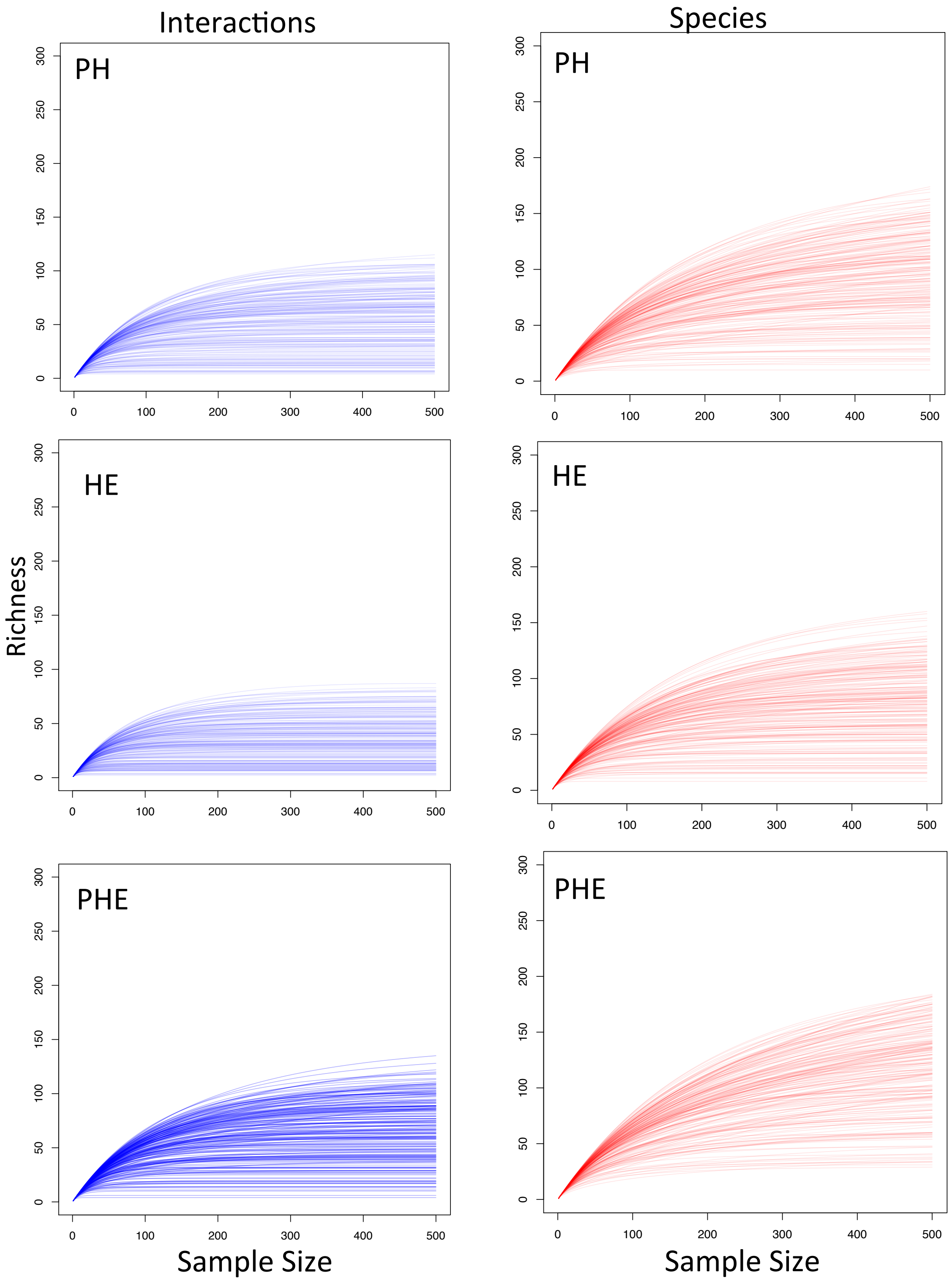

Interactions
Species
PH
HE
PHE
Richness
Sample Size
Sample Size
0
50
100
150
200
250
300
0
100
200
300
400
500
